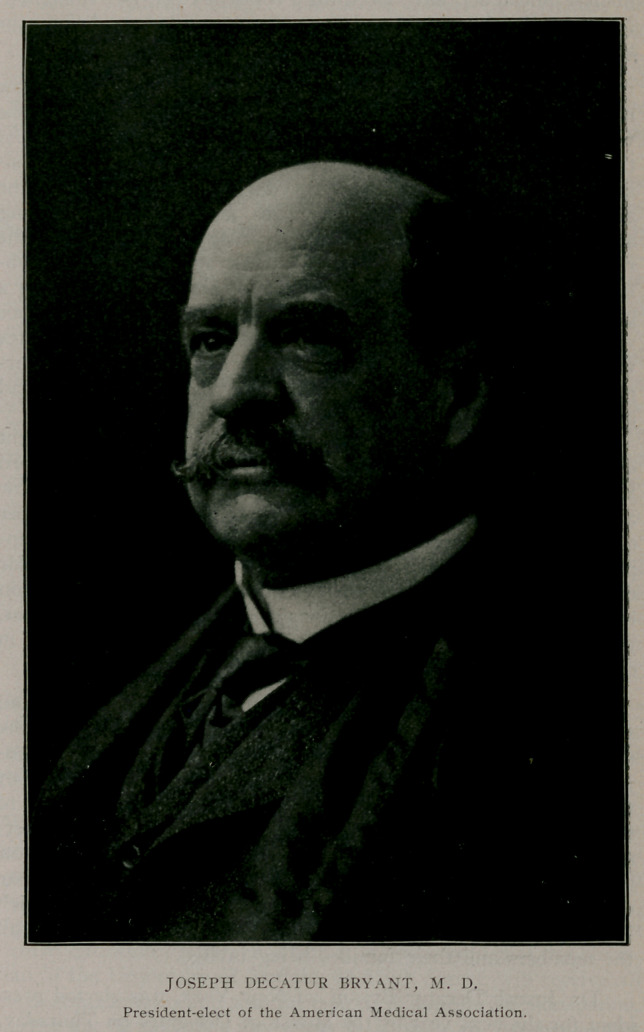# Fifty-Seventh Meeting of the A. M. A.—1906

**Published:** 1906-08

**Authors:** 


BUFFALO MEDICAL JOURNAL.
A Monthly Review of Medicine and Surgery.

*EDITOR:*
WILLIAM WARREN POTTER, M. D.

All communications, whether of a literary or business nature, books for review and exchanges, should be addressed to the editor 284 Franklin St., Buffalo, N. Y.

Vol. LXII. AUGUST, 1906. No. 1

### Fifty-Seventh Meeting of the A. M. A.—1906.

THE Boston meeting of the American Medical Association will be remembered long and pleasantly by all who were so fortunate as to attend, because of many of its sepecial features and for the excellence of its scientific work. The weather during the entire week was favorable to all the functions and work, whether held indoors or on the lawns, and for riding or driving it could not have been more propitious. Good weather is an important factor in promoting a successful gathering of men and women in such large numbers as attended at Boston.

This brings us to remark that the registry was much larger than ever before, reaching the aggregate of plus 4,700, which means a probable attendance of at least 6,000 persons including families, foreign, other guests and exhibitors. The meeting, therefore, was probably the largest medical gathering the world has ever witnessed. The cause is not difficult to determine. In the first place, Boston is an attractive city, full of historic interest, a city of culture, art, refinement and charming people. Again, the management of the details of the meeting was superb. The chairman of the committee of arrangements, Dr. Herbert L. Burrell, and his capable staff opened the campaign many months beforehand and pursued it with energy until the meeting was over. The large influx of people took their appointed places at the several hotels and boarding houses without friction, while all the social functions were arranged with a completeness that could only follow most careful preparation and thorough discipline.

The House of Delegates performed an astonishing amount of work and avoided serious conflict in discussing the several divergent interests of a large and rapidly increasing constituency, adjourning with a consolidated and improved condition of the profession as the result of its arduous legislative work. President McMurtry in his address at the opening session of the house called attention, inter alia, most felicitously to the fact that for the first time in twenty-five years the medical profession of the state of New York was represented in the councils of the American Medical Association. "During all this period," said he, "the schism in this state had prevented representation from the very society which took the initiative in founding this national association." Dr. McMurtry's words, which were in the nature of a welcome to the delegates from the Medical Society of the State of New York, representing a reunited profession in the Empire state, were received with hearty applause.

The President made several valuable recommendations for the improvement of medical conditions, one of the most important of which related to the manner of voting for trustees. Heretofore, it has been the practice to vote for the three candidates to fill vacancies occurring each year in one group, whereas now, under the wise suggestion of Dr. McMurtry, a separate ballot for each trustee must be voted.

The ceremonies opening the general session on Tuesday were more elaborate than ever before. After the large auditorium of Mechanics Hall was filled, the procession which had formed in the banquet hall, led by the band of the first corps of cadets, marched up the aisle amid the plaudits of the vast assemblage. The marshal was Dr. Paul Thorndike, his chief aide being Dr. T. L. Wilson, and his staff was augmented by a large corps of assistants, all being the younger members of the profession. Preceded by a group of policemen and following the band, Governor Curtis Guild, Jr., and the Reverend Edward Everett Hale led the column. Then followed Dr. Herbert L. Burrell, chairman of the committee of arrangements, escorting Mayor Fitzgerald; next came the officers of the association led by President McMurtry and President-elect Mayo, and after these the foreign guests each escorted by an ex-president or other prominent member of the association. These with a few invited guests, among whom were Drs. S. Weir Mitchell and Alonzo Garcelon, made up a stage group of imposing presence.

Dr. Lewis S. McMurtry, the retiring president, acted as master of ceremonies and after a few words of greeting he introduced the Reverend Dr. Hale, who pronounced the invocation. Governor Guild was then introduced and was given a most cordial greeting by the great audience. The Governor's speech of welcome was one which may well serve as a model of eloquence and patriotic utterance for similar occasions. It deserves to be preserved in the history of the Boston meeting. Next after the Governor, Dr. McMurtry introduced President Eliot of Harvard University, who, with befitting words, welcomed the association in the name of the institution over which he presides.

Dr. Richard T. Cabot, President of the Massachusetts State Medical Society, who brought the greetings of that body, was next introduced and welcomed the American Medical Association in behalf of the medical profession of the commonwealth of

WILLIAM J. MAYO, M. D.

President of the American Medical Association.

Massachusetts. Next after Dr. Cabot, the president introduced Mayor Fitzgerald, whose address of welcome on behalf of the city of Boston, was manly, eloquent, classic and in every way befitting the occasion. After the mayor came Dr. Burrell,

JOSEPH DECATUR BRYANT, M. D.

President-elect of the American Medical Association.

who, as chairman of the committee of arrangements, gave the usual budget of information and then President McMurtry, as his last official act, introduced the President-elect, Dr. William J. Mayo, of Rochester, Minnesota, who proceeded to deliver the annual address.

Dr. Mayo's personality is an interesting one. Slightly below medium stature, with hair tinged with grey,—prematurely so one would say,—he stands erect and looks straight into the eye of the person with whom he talks, speaking earnestly and interestingly, without circumlocution but with intelligence. He has the air of a man unaccustomed to leisure, and takes up one thing after another as presented, not impatiently but reaching for the second just as the first is finished. His address, already published in the journals, is a state paper of importance, showing a grasp of American medical affairs, and in intimate acquaintance with medical men. He made a hit in the house of delegates when, after some of the members had presented plaints, he surprised and amused the house by stating that if any others had grievances then was the time to present them.

The scientific work of the several sections was of excellent quality, but some of the programs were too crowded to afford justice to all the papers. If the association continues to grow at the present rate something must be done to limit the number of papers, or to increase the number of sessions. It would appear to be a good plan to increase the number of days to five for each anual meeting. If this were done and great discrimination exercised by the officers of sections in preparing their programs, much valuable time could be saved for the discussions which in consequence could be given wider range.

The social features of the meeting were conducted on a grand scale. The public receptions were superbly managed, and the numerous private dinners and luncheons added much to the enjoyment and recreation of the members and visitors. Two of these deserve special mention,—one being Dr. Mayo's dinner to the foreign guests on Monday evening, which was simply perfect in all its details; the other consisted of a series of afternoon teas on the terraces of the Harvard University grounds at four o'clock Tuesday, Wednesday, and Thursday, at each of which Dr. John Collins Warren and Mrs. Roger Wolcott received, all the members and their families being invited.

Dr. Joseph Decatur Bryant, of New York, was elected president for the next year by the House of Delegates on Thursday, and it is difficult to say how this important duty could have been performed in a more satisfactory fashion. Dr. Bryant has been identified prominently with American medicine and medical affairs for more than twenty-five years, during which time he has taught either anatomy or surgery in what is now University and Bellevue Medical College. He is the author of a treatise on surgery in two volumes which is used as a textbook in our leading medical colleges, and also is the senior editor of the American Practice of Surgery, a work in eight volumes, the first of which has just been published, and which is designed to be the most comprehensive surgical treatise ever issued from the press, in this or any other country.

Dr. Bryant is the reigning president of the Medical Society of the State of New York, serving his second term and was conspicuous in promoting the unification of the medical profession in the Empire state and, as a leading member of the joint committee, did as much, perhaps, as any other member to restore harmony through the amalgamation of the two state medical organisations so happily accomplished last year.

It is more than probable that the national association had this fact in mind in inviting Dr. Bryant to the presidency so soon after the chair had been held by another distinguished New Yorker. Be that as it may, the new president takes office at the right time to further solidify the bands of unity that he so capably helped to forge during all the years of arduous committee work in relation to amalgamation.

---

If any of our readers are interested in automobiles, all such will find a card on page x of our advertising columns offering for sale a Pierce Stanhope in perfect order and at a low price.

---

Attention is invited to the advertisement of the Buffalo Electro-Mechanical Company on advertisement page xx. Physicians in need of supplies or repairs relating to electro-therapeutic instruments, or to any electrical work, will do well to call on this company.

---

We have called attention heretofore to the needs of San Francisco physicians in regard to materials for office use, such as instruments, books, clinical thermometers and the like. Last month we published a letter from Dr. Charles G. Stockton, of this city, on the subject. Again we make the appeal and invite attention to Dr. Stockton's letter, in which it is announced that the librarian of the University of Buffalo, 24 High Street, will receive all such contributions and ship them to their appropriate destination. Let it not be said that physicians in Buffalo are derelict in this important duty.

## Figures and Tables

**Figure f1:**
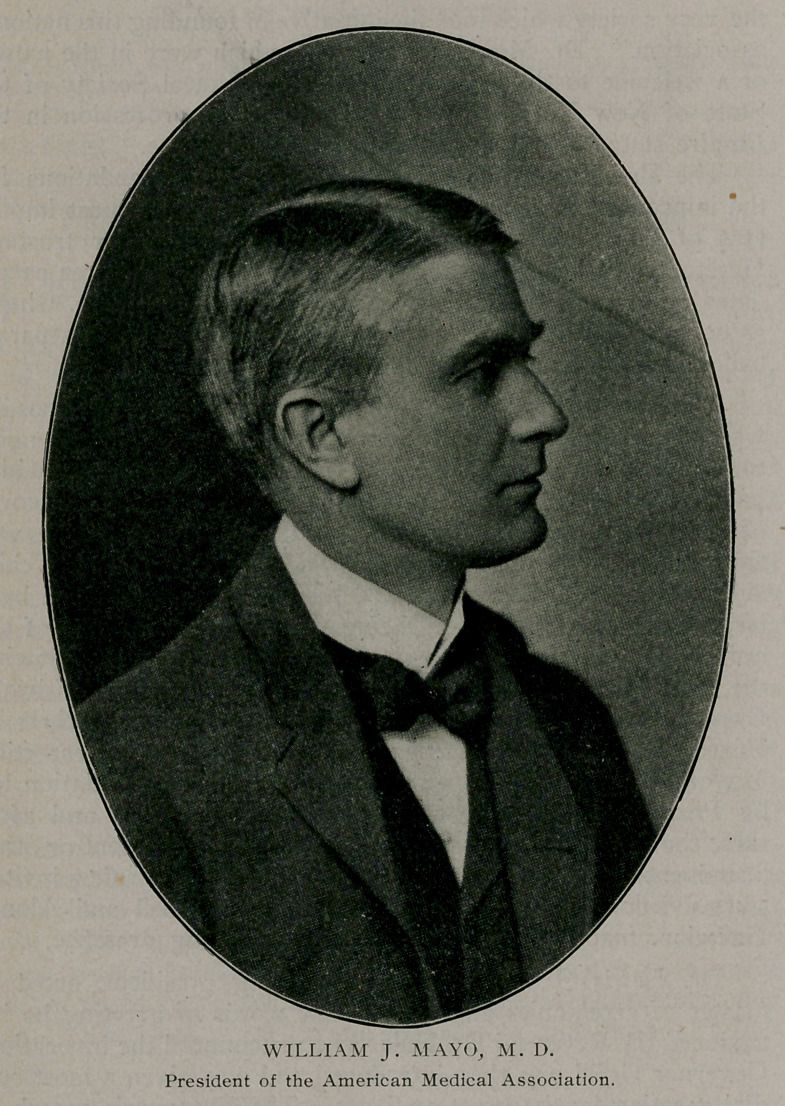


**Figure f2:**